# Intravenous Infusion of Magnesium Sulphate During Subarachnoid Anaesthesia in Hip Surgery and Its Effect on Postoperative Analgesia: Our Experience

**Published:** 2013-01-04

**Authors:** A Pastore, M Lanna, N Lombardo, C Policastro, C Iacovazzo

**Affiliations:** Department of Anaesthesia and Intensive Care, University of Naples “Federico II”

**Keywords:** Magnesium Sulphate, Spinal Anaesthesia, Hip Arthroplasty, Postoperative Pain, Postoperative Analgesia

## Abstract

The treatment of degenerative hip joint disease involves modern operative techniques and the use of prosthetic devices individualized on each patient. Being a surgery of considerable importance, great attention is always given by the anaesthesiologist to postoperative analgesia. In general, our goal is to limit the doses of NSAIDs, known to be associated with haemostasis interference and alteration of gastrointestinal apparatus; component of our baseline analgesic protocols after arthroplasty is morphine given parenterally. In order to steadily improve analgesic techniques, which directly impact on patient outcome, we experimented the use of a continuous infusion of magnesium sulphate during subarachnoid anaesthesia. Magnesium sulphate is the drug of choice in case of eclampsia, and pre-eclampsia (for the risk of evolution in eclampsia). According to the most recent findings, this drug has also analgesic properties: its use as an adjunct to analgesia is based on a non-competitive antagonism towards the NMDA receptor and on the blocking of calcium channels: these properties prevent the mechanisms of central sensitization due to nociceptive stimulation of peripheral nerves.

## I. INTRODUCTION

The treatment of degenerative hip joint disease involves modern operative techniques and the use of prosthetic devices individualized on each patient [1,2,3].

In recent years the hip surgery has evolved: the surgeon has increasingly refined surgical techniques that allow him to safely perform hip arthroplasty without altering anatomical structures. The indications have expanded, the average age of patients undergoing surgery has reduced, while life expectancy has considerably increased, thus creating to the surgeon problems sometime difficult to solve [4].

The aim of the intervention of hip replacement is to recreate a not painful, stable joint, which allows the patients to carry out their daily activities, but it must last over time and determine no biological intolerance. The hip surgery can be classified into three types: the total replacement or arthroplasty, in which the surgeon intervene on both articular components, femoral and acetabular; partial replacement, commonly indicated with the term endoprosthesis, reserved for the treatment of medial fractures of the neck of the femur, which allows to preserve the acetabulum; the revision, which is the replacement of a previously implanted device.

The diseases that may require hip arthroplasty can be divided into elective indication pathologies (primary arthrosis and secondary trauma in advanced stages, rheumatoid arthritis, aseptic osteonecrosis of the femoral head, hip dysplasia, ankylosing spondylitis, psoriatic arthritis, fracture of the medial femoral neck at the age of 60 years, secondary tumour lesions), and indication (juvenile rheumatoid arthritis, Paget’s disease, bone tumours primitive medial femoral neck fracture in aged less than 60 years, bone tuberculosis, osteomyelitis, results of arthrodesis, dysplasia, acetabular fractures).

All these causes alter the structures of the hip causing disability with pain, impotence and lameness. Being a surgery of considerable importance, great attention is always given by the anaesthesiologist to postoperative analgesia: in this field, the knowledge is constantly evolving, as evidenced in literature [5].

In general, our goal is to limit the doses of NSAIDs, known to be associated with haemostasis interference and alteration of gastrointestinal apparatus; component of our baseline analgesic protocols after arthroplasty is morphine given parenterally.

In order to steadily improve analgesic techniques, which directly impact on patient outcome, we experimented the use of a continuous infusion of magnesium sulphate during subarachnoid anaesthesia [6,7].

Magnesium sulphate is the drug of choice in case of eclampsia [8–10] (for the prevention of recurrent seizures), and pre-eclampsia (for the risk of evolution in eclampsia). According to the most recent findings [11,12], this drug has also analgesic properties: its use as an adjunct to analgesia is based on a noncompetitive antagonism towards the NMDA receptor [13] and on the blocking of calcium channels [14]: these properties prevent the mechanisms of central sensitization due to nociceptive stimulation of peripheral nerves [15].

The purpose of this study was to evaluate the effect of the intravenous infusion of magnesium sulphate during spinal anaesthesia on the quality of analgesia and consumption of analgesics in the first 24 h after hip replacement surgery.

## II. METHODOLOGY

This controlled-randomized, double-blind trial was conducted in the months of January, February, March 2012, on 12 patients referred to the Department of Orthopaedics and Traumatology of our AOU, who had an indication to perform hip replacement in spinal anaesthesia. These patients were informed of the current study and was collected their informed consent. It was also explained to them the proper functioning of the VAS (visual analogue scale of 10 cm where 0 cm = no pain, 10 cm = worst pain imaginable).

Inclusion criteria: patients ASA I–II, aged up to 70 years, candidates for elective total hip replacement. Exclusion criteria: heart diseases, hypermagnesaemia, known hypersensitivity to magnesium sulphate, previous treatment with calcium channel blockers, all types of heart block, no predictors of intermediate or higher cardiac risk, acute or chronic kidney failure, liver or neuromuscular disease, abuse of opioids or analgesics.

The anthropometric parameters of the patients were: 1.70 m10 cm of height, 70 kg ± 20kg of weight in the two groups, the mean values were similar (1.68 m and 73kg MS group, group SI 1.72 m and 68 kg). Given the strict criteria of inclusion and exclusion, the groups were found to be homogeneous.

The data were processed at the end of the study, calculating mean and standard deviation of the VAS and analgesic consumption, then compared by statistical analysis with T-student test (significance level p <0.01).

Patients were randomized into 2 groups of 6 elements each one: all patients received premedication with midazolam 0.03 mg / kg, then we performed spinal anaesthesia in the sitting position at the level L2/L3 with a spinal 27 gauge Whitacre needle, using hyperbaric bupivacaine 0,5%;standard monitoring, ECG/SpO2 and non-invasive blood pressure, were carried out.

All patients received 500 ml Ringer’s lactate solution in 15 minutes, to counteract hypotension and the possible negative inotropic effect of the bolus magnesium sulphate.

Immediately after the injection of the local anaesthetic, GROUP MS received 50 mg / kg of MgSO4 in 15 min and then 15 mg / kg / h until the end of the surgical procedure, while the control group received the same volume of isotonic saline for the same period of time.

In the case of bradycardia (HR lower than 45 bpm) atropine was administered in boluses of 0.2 mg; in case of hypotension (SBP lower than 90 mmHg or 20% decrease of the baseline value) 5 mg of ephedrine iv were given.

The average time of the procedure was 100 minutes (60 min–120 min). It has never been necessary to convert the surgery under general anaesthesia.

After the surgical procedure 90 mg of ketorolac in 24 hours via iv elastomeric infusor at the rate of 2ml / h were administered to both groups. If necessary (VAS 6 or more), paracetamol 1 g iv. was administered as an analgesic rescue in the postoperative period.

We subsequently evaluated for 24 h the following parameters:

-Pain (VAS) at the time T1 (4h), T2 (12h), T3 (24h).
-Consumption of analgesics rescue.
-Incidence of side effects such as shivering, nausea, vomiting.
-Surgical complications: bleeding and bruising, signs of infection.
-Concentrations of serum magnesium were checked before practicing anaesthesia, immediately after surgery, and at 1 h and 24 h after surgery (normal values at our laboratory services = 0.75 to 1 mmol / L).

## III. RESULTS

In case of VAS 6 paracetamol 1 gr iv was administered. The postoperative pain scores were significantly lower in MS group at 4 h, 12 h, 24 h after surgery (p <0.01), the consumption of rescue analgesics 12 h and 24 h after surgery was also significantly decreased (p <0.01) .

No patient of the MS group requires analgesic drugs in the first 4 hours, one patient needed paracetamol at time T2 and one at time T3. In SI group we administered analgesic drugs one time in the first 4 h, 4 times in 12 hours and 5 times in 24 hours.

The serum concentration of MgSO4 has never exceeded 1 mmol / L; moreover no side effect of hypermagnesemia occurred.

The hemodynamic changes, the incidence of PONV and shivering, the recovery of the motility of the lower limbs were similar in both groups and were not statistically significant (p>0,05).

## IV. DISCUSSION

The postoperative pain after hip replacement is usually described as moderate to severe, thus affecting patient outcome [13,14]. Although the mechanism is not completely understood, magnesium sulphate could prevent central sensitization following peripheral nociceptive stimulation, and abolish hypersensitivity. This analgesic properties are due to its action on NMDA receptors and calcium channels.

The perioperative administration of magnesium sulphate [17,18], either in single injection or as a continuous infusion, appears to be effective in reducing postoperative pain and it is associated with minor consumption of analgesic drugs in the first 24 hours, without significant side effects.

## V. CONCLUSION

Although carried out on a limited number of patients, this study confirms that the iv infusion of magnesium sulphate during spinal anaesthesia improves the quality of analgesia and reduces the postoperative consumption of analgesics. This property is probably related to the competitive antagonism on NMDA receptor and to the blockade of calcium channels, both involved in the mechanisms of central sensitization of pain. Magnesium sulphate appears to be safe, free from side effects and well tolerated at low dosage. These results could provide a basis for further investigation about the use of this drug as an important analgesic adjuvant in the perioperative period, especially in patients in which opiates or NSAIDs are contraindicated.

## Figures and Tables

**Fig.1.**
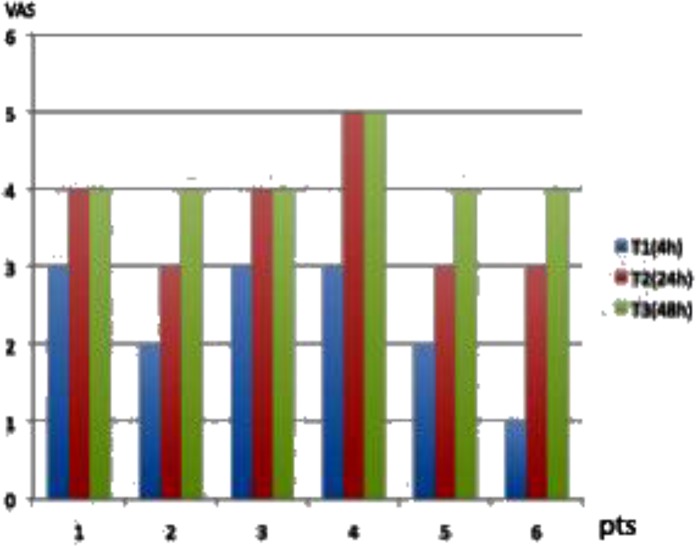
VAS of MS group patients

**Fig. 2.**
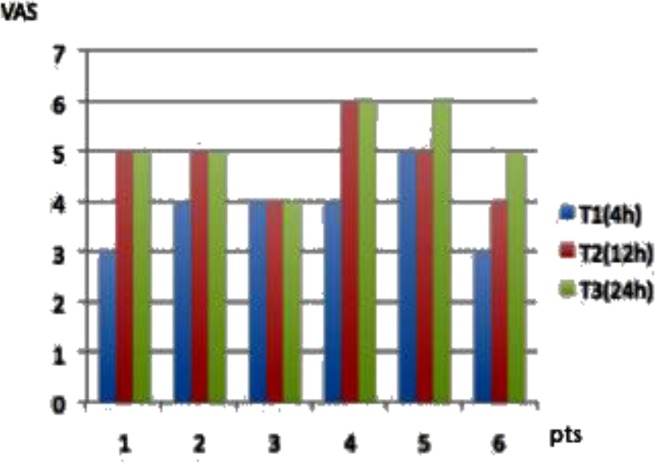
VAS of SI group patients
